# A Case of Type 2 Amiodarone-Induced Thyrotoxicosis That Underwent Total Thyroidectomy under High-Dose Steroid Administration

**DOI:** 10.1155/2015/416145

**Published:** 2015-01-13

**Authors:** Koshi Hashimoto, Masaki Ota, Tadanobu Irie, Daisuke Takata, Tadashi Nakajima, Yoshiaki Kaneko, Yuko Tanaka, Shunichi Matsumoto, Yasuyo Nakajima, Masahiko Kurabayashi, Tetsunari Oyama, Izumi Takeyoshi, Masatomo Mori, Masanobu Yamada

**Affiliations:** ^1^Department of Medicine and Molecular Science, Gunma University Graduate School of Medicine, 3-39-22 Showa-machi, Maebashi, Gunma 371-8511, Japan; ^2^Department of Preemptive Medicine and Metabolism, Graduate School of Medical and Dental Sciences, Tokyo Medical and Dental University, 1-5-45 Yushima, Bunkyo-ku, Tokyo 113-8510, Japan; ^3^Department of Medicine and Biological Science, Gunma University Graduate School of Medicine, 3-39-22 Showa-machi, Maebashi, Gunma 371-8511, Japan; ^4^Department of Thoracic Visceral Organ Surgery, Gunma University Graduate School of Medicine, 3-39-22 Showa-machi, Maebashi, Gunma 371-8511, Japan; ^5^Department of Diagnostic Pathology, Gunma University Graduate School of Medicine, 3-39-22 Showa-machi, Maebashi, Gunma 371-8511, Japan

## Abstract

Amiodarone is used commonly and effectively in the treatment of arrhythmia; however, it may cause thyrotoxicosis categorized into two types: iodine-induced hyperthyroidism (type 1 amiodarone-induced thyrotoxicosis (AIT)) and destructive thyroiditis (type 2 AIT). We experienced a case of type 2 AIT, in which high-dose steroid was administered intravenously, and we finally decided to perform total thyroidectomy, resulting in a complete cure of the AIT. Even though steroid had been administered to the patient (maximum 80 mg of prednisolone), the operation was performed safely and no acute adrenal crisis as steroid withdrawal syndrome was found after the operation. Few cases of type 2 AIT that underwent total thyroidectomy with high-dose steroid administration have been reported. The current case suggests that total thyroidectomy should be taken into consideration for patients with AIT who cannot be controlled by medical treatment and even in those under high-dose steroid administration.

## 1. Introduction

Amiodarone, a benzofuranic acid derivative, is a potent class III antiarrhythmic drug that is used in the treatment of paroxysmal supraventricular tachycardia, malignant ventricular tachyarrhythmia, atrial flutter, and fibrillation [1]. It is an iodine-rich (37% of its weight) compound with a molecular structure similar to thyroxine (T_4_) and triiodothyronine (T_3_). It is also a fat-soluble drug with a long half-life (107 days), which allows the effects to be seen months after discontinuation [2]. Conventional doses of 100 to 600 mg of amiodarone per day provide 37 to 222 mg of organic iodine, which is up to 50–100 times the optimal daily iodine intake, and greatly expand the systemic and thyroidal iodine pools [3]. Although it may reduce cardiac-related mortality and improve survival rates, amiodarone can also cause the development of serious thyroid dysfunction in patients with or without underlying thyroid disease [4, 5]. The rate of occurrence of thyroid dysfunction, either thyrotoxicosis (amiodarone-induced thyrotoxicosis: AIT) or hypothyroidism, is 15–20% [6]. AIT is more prevalent in iodine-deficient areas and is currently known to be catabolized by two mechanisms: iodine-induced hyperthyroidism (type 1 AIT) and destructive thyroiditis (type 2 AIT), caused by amiodarone itself and its high iodine content. Type 1 AIT develops in subjects with underlying thyroid disease and is exacerbated by iodine loading of thyroid autonomous function; on the other hand, type 2 AIT occurs in patients with no history of thyroid disease and is probably consequent to drug-induced destructive thyroiditis. Moreover, the two mechanisms may occur in the same patient (mixed type) [4, 7]. AIT may develop early during amiodarone treatment or even several months after it has been discontinued. This is due to the fact that amiodarone and its metabolites have a long half-life and are stored in various tissues, particularly in fat, from which they are released very slowly. The onset of AIT is often sudden and explosive [8]. AIT worsens ventricular arrhythmia because of the hyperthyroid state. Medical management including steroid administration against AIT may produce a temporary response but often fails to resolve the thyrotoxicosis [9]. Here, we experienced a case of type 2 AIT, in which high-dose steroid was administered intravenously, and we finally decided to perform total thyroidectomy, resulting in complete cure of the AIT. Even though steroid had been administered to the patient (maximum 80 mg of prednisolone: PSL), the operation was performed safely and no acute adrenal crisis as steroid withdrawal syndrome was found after the operation. Few cases of AIT with steroid administration that underwent total thyroidectomy have been reported. The current case suggests that total thyroidectomy should be taken into consideration for patients with AIT who cannot be controlled by medical treatment and even in those under steroid administration.

## 2. Case Presentation

**Figure 1:**
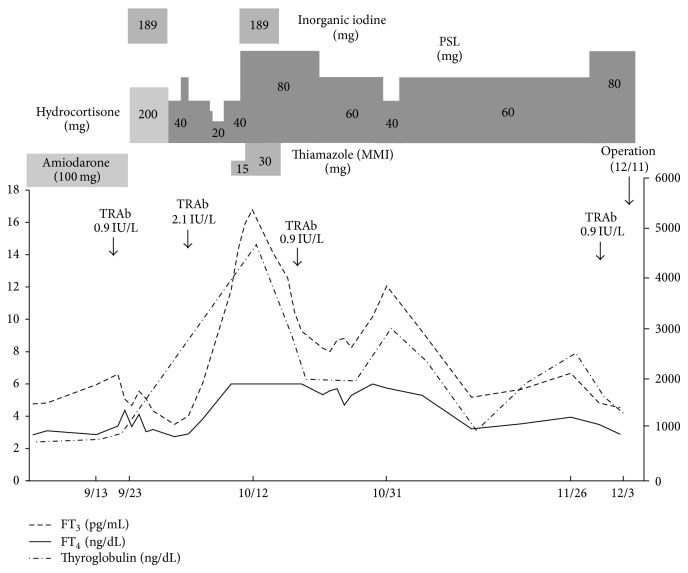
Clinical course of the case.

**Figure 2:**
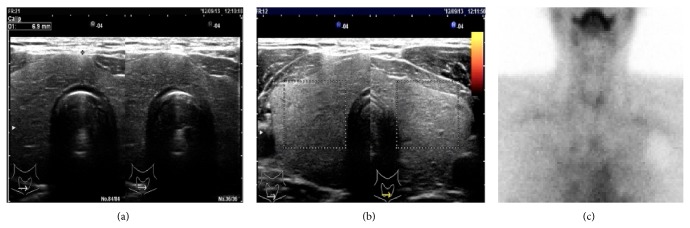
(a) Ultrasonic study of the thyroid revealing mild swelling with 6.9 mm isthmus diameter. (b) Doppler flow study of the thyroid gland revealing no blood flow increase. (c) Thyroid ^99m^Tc scintigraphy revealing no uptake.

**Figure 3:**
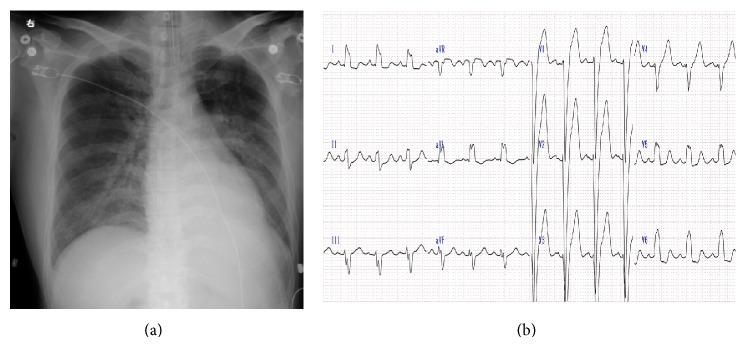
(a) Chest X-ray on admission demonstrating severe cardiomegaly. (b) Electrocardiogram on admission showing wide QRS pattern and tachycardia.

**Figure 4:**
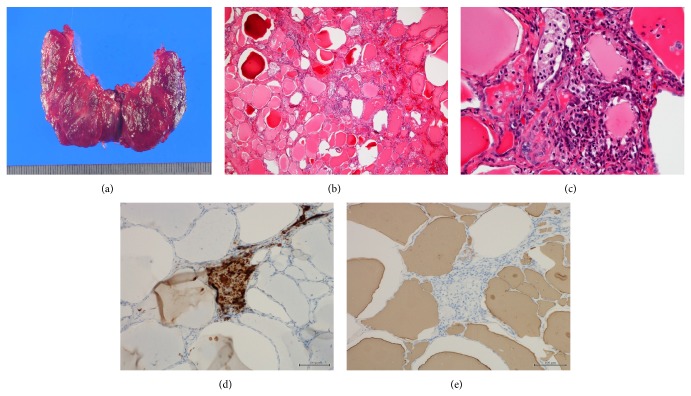
(a) Gross pathological findings of the excised thyroid gland. (b, c) H-E staining of the thyroid gland in low-power (b) and high-power fields (c). Several sizes of follicle were regularly lined with flattened follicular epithelium. The lumen was filled with colloid. Scattered disrupted follicles with enlarged epithelium and cytoplasmic vacuoles were observed (b). Macrophages had infiltrated and multinucleated giant cells were found in follicular lumen (c). Immunostaining with anti-KP-1 (CD68) antibody (d). Immunostaining with antithyroglobulin antibody (e).

A 40-year-old man suffering from dilated cardiomyopathy had been prescribed amiodarone for 2.5 years. Seven weeks before the consultation at our department, his serum-free T_4_ levels increased above the upper limit and thyrotoxicosis developed. His thyroid status was as shown in Figure 1. An attending cardiologist consulted at our thyroid clinic about the patient's thyrotoxicosis, but he had no complaints. He did not show any tachycardia or finger tremor, despite the thyrotoxicosis. His thyroid gland was not swollen and ultrasonic study revealed a slightly enlarged thyroid gland with almost monotonous echogenicity (Figure 2(a)). The Doppler flow rate inside the thyroid gland was not increased (Figure 2(b)). To differentiate the diagnosis of thyrotoxicosis, we planned to investigate thyroid iodine uptake. Ten days after the first visit, he showed symptoms of acute heart failure and was admitted to the intensive care unit of our hospital. His thyrotoxicosis had worsened by the time of admission, with increased levels of thyroglobulin, suggesting destructive thyroiditis (Table 1). Amiodarone administration was stopped and inorganic iodine administration (189 mg/day) was started upon admission; however, his thyrotoxicosis was prolonged and worsened. His cardiac function also worsened, with the thyrotoxicosis being exacerbated (Figure 3). On admission, his heart rate was over 180 bpm and systolic blood pressure was 220 mmHg. Oxygen saturation rate was 70% under 10 L/min of oxygen administration with a venturi mask. Intra-arterial balloon pumping was performed to maintain the circulation. On the day after admission, administration of 200 mg of hydrocortisone was started, in addition to inorganic iodine. After the hydrocortisone administration, free T_3_ levels were somewhat improved, but free T_4_ levels remained high. To control and suppress the destruction of the thyroid, 40 mg of PSL was administered instead of hydrocortisone. Subsequently, 60 mg of PSL improved the serum-free T_4_ levels, so we tapered the dose of PSL gradually. However, at a dose of 20 mg of PSL, the thyrotoxicosis relapsed. At this point, TSH receptor antibody (TRAb) became positive (Figure 1), so we decided to prescribe 15 mg of methimazole (MMI) together with 40 mg of PSL. Two days after these prescriptions, his free T_4_ levels increased to above the normal range. Thirty milligrams of MMI, 40 mg of PSL, and inorganic iodine (189 mg/day) did not suppress the destructive thyroiditis. On the 17th day of admission, thyroid ^99m^Tc uptake was investigated, but none was observed (Figure 2(c)). At this point, we made a final diagnosis of type 2 amiodarone-induced thyrotoxicosis (AIT). On the 23rd day of admission, MMI was discontinued and the administration of 80 mg of PSL was maintained. Subsequently, we attempted to taper the dose of PSL, but under a dose of 80 mg of PSL, overt thyrotoxicosis was not controlled (Figure 1). Since over 2.5 months had passed since a high dose of PSL had been administered, we decided to perform total thyroidectomy. The administration of 80 mg of PSL was continued until the operation. With informed consent from the patient and his wife, total thyroidectomy was performed on the 78th day of admission. Intravenous administration of 40 mg of PSL and 200 mg of hydrocortisone was performed during the operation. The operation was safely performed and 25.6 g of thyroid was resected. After the operation, PSL was discontinued and the dose of hydrocortisone was carefully tapered. Two days after the thyroidectomy, hydrocortisone was tapered to 100 mg and administered orally. Then, hydrocortisone was again gradually tapered to 15 mg eleven days after the surgery. Twenty-five days after the operation, hydrocortisone was tapered to 5 mg, and it was discontinued on the forty-sixth day after the thyroidectomy. During the tapering of hydrocortisone and after its discontinuation, the patient demonstrated no symptoms of adrenal insufficiency. Pathological findings of the excised thyroid gland are as shown in Figure 4. Grossly, the lobes became firm in consistency but maintained their normal shape (Figure 4(a)). On microscopy, several sizes of follicles were regularly lined with flattened follicular epithelium. The lumen was filled with colloid. Scattered disrupted follicles with enlarged epithelium and cytoplasmic vacuoles were observed (Figure 4(b)). It is of note that macrophages had infiltrated and multinucleated giant cells were also found in the follicular lumen (Figure 4(c)). Immunostaining with anti-KP1 (CD68) and antithyroglobulin antibodies confirmed that the infiltrated cells were macrophages but not follicular cells (Figures 4(d) and 4(e)). These findings characterized by scattered follicle disruption, vacuoles in epithelial cells, and macrophage infiltration are compatible with amiodarone toxicity [10].

After the operation, the patient's thyrotoxicosis rapidly disappeared and the thyroid function was normalized with 100 *μ*g of levothyroxine (L-T_4_). Thirty-six days after the thyroidectomy, implantation of a left ventricular epicardial lead was performed under the administration of 5 mg of hydrocortisone. We administered 200 mg of hydrocortisone intravenously during the procedure and the implantation was performed safely. After the implantation, his cardiac function was dramatically improved. On the 130th day of admission, the administration of hydrocortisone was discontinued and he was discharged from the hospital on foot.

## 3. Discussion

We have experienced a severe case of type 2 AIT, which was uncontrollable with high-dose PSL. The final diagnosis was difficult since TRAb was positive at one time in the clinical course, which led us to consider that this case may be type 1 and type 2 mixed AIT. Therefore, we administered MMI to the patient at some points in the clinical course. However, taken together with the findings from a thyroid scan and laboratory data, this case should be classified as type 2 AIT, even though it has been reported that the features of hyperthyroidism and destructive thyroiditis may concomitantly be present. Thionamides such as methimazole and propylthiouracil are not effective in type 2 AIT [7]. It was a very difficult decision to perform the total thyroidectomy since a maximum of 80 mg of PSL had been administered. However, considering the side effects, including infection, of long-term use of high-dose steroid, we did not have an alternative approach other than thyroidectomy. Moreover, in view of his cardiac status, implantation of a left ventricular epicardial lead needed to be performed as soon as possible.

Type 2 AIT may be self-limiting, and some reports recommend continuation of amiodarone for the cardiac effect [11]. Steroid is the best treatment for type 2 AIT [12]. As other treatments, the use of lithium, potassium perchlorate, and iopanoic acid has been proposed for type 2 AIT, but the evidence is too limited to support their effectiveness [7]. Plasmapheresis can provide acute relief from type 2 AIT but is not generally used because of its transient effects, its cost, and the impossibility of maintaining its use over the long term [5, 7]. In addition, radioactive iodine therapy is in principle not feasible in type 2 AIT patients because iodine uptake is usually suppressed, as shown in this case [5, 7]. The initial PSL dose is about 0.5–0.7 mg/kg body weight per day and the treatment is usually continued for 3 months [6]. The current case can be considered rare because a maximum of 80 mg per day of PSL was required to control the thyrotoxicosis. Therefore, we were very careful to taper the dose of steroid after the total thyroidectomy and the tapering was performed successfully. Total thyroidectomy with general anesthesia is not the first-line treatment for type 2 AIT, since there may be potential risks, such as severe arrhythmia, in the perioperative period in these patients with underlying cardiac disorders [7]. However, this approach may be required in patients who are resistant to medical treatments [5, 9, 13, 14]. Minimally invasive thyroidectomy with local anesthesia may further reduce the risk [15]; however, its use has not yet spread widely.

Thyroidectomy is an efficacious approach for type 2 AIT patients who are resistant to high-dose PSL to control thyrotoxicosis. Physicians should not be reluctant to make a decision to perform the surgery and total thyroidectomy can be performed more safely than expected, even if high-dose PSL has been administered to the patients.

## Figures and Tables

**Table 1:** Laboratory data on admission.

Hematology		Biochemistry		Cardiology		
RBC	494 × 10^4^/*μ*L	T.P.	6.6 g/dL	Troponin I	0.53 ng/mL	(<0.1)
Hb.	16.4 g/dL	Alb.	3.7 g/dL	BNP	579.0 pg/mL	(0–18.4)
Ht.	47.4%	T.Bil.	0.8 mg/dL	**Thyroid function**		
WBC	16100/*μ*L	GOT	115 IU/L	TSH	<0.05 *μ*U/mL	(0.35–4.94)
Plt.	27.8 × 10^4^/mL	GPT	138 IU/L	Free T4	3.39 ng/dL	(0.70–1.48)
Fib.	372 mg/dL	LDH	356 IU/L	Free T3	6.61 pg/mL	(1.71–3.71)
PT	93%	ALP	223 IU/L	Thyglobulin	1025.0 ng/mL	(0–32.7)
APTT	27.8 sec	*γ*-GTP	310 IU/L	TGHA	<100X	
FDP	13.6 mg/dL	AMY	293 IU/L	MCHA	<100X	
		LDL-Cho	136 mg/dL	TRAb	0.9 IU/L	(<1.0)
		UA	5.1 mg/dL			
		Glu	226 mg/dL			
		CRP	0.23 mg/dL			
		BUN	24 mg/dL			
		Cr	0.89 mg/dL			
		Na	139 mEq/L			
		K	5.1 mEq/L			
		Cl	105 mEq/L			

## References

[1] Roy D., Talajic M., Dorian P. (2000). Amiodarone to prevent recurrence of atrial fibrillation. *New England Journal of Medicine*.

[2] Zipes D. P., Prystowsky E. N., Heger J. J. (1984). Amiodarone: electrophysiologic actions, pharmacokinetics and clinical effects. *Journal of the American College of Cardiology*.

[3] Rao R. H., McCready V. R., Spathis G. S. (1986). Iodine kinetic studies during amiodarone treatment. *Journal of Clinical Endocrinology and Metabolism*.

[4] Piga M., Serra A., Boi F., Tanda M. L., Martino E., Mariotti S. (2008). Amiodarone-induced thyrotoxicosis: a review. *Minerva Endocrinologica*.

[5] Franzese C. B., Stock B. C. (2002). Amiodarone-induced thyrotoxicosis: a case for surgical management. *American Journal of Otolaryngology—Head and Neck Medicine and Surgery*.

[6] Martino E., Bartalena L., Bogazzi F., Braverman L. E. (2001). The effects of amiodarone on the thyroid. *Endocrine Reviews*.

[7] Bogazzi F., Bartalena L., Martino E. (2010). Approach to the patient with amiodarone-induced thyrotoxicosis. *Journal of Clinical Endocrinology and Metabolism*.

[8] Conen D., Melly L., Kaufmann C. (2007). Amiodarone-induced thyrotoxicosis: clinical course and predictors of outcome. *Journal of the American College of Cardiology*.

[9] Birkedal C., Touliatos J., Gaskin T., Spence R. K. (2001). Surgical considerations for treatment of amiodarone-induced thyrotoxicosis. *Current Surgery*.

[10] Nakazawa T., Murata S.-I., Kondo T. (2008). Histopathology of the thyroid in amiodarone-induced hypothyroidism. *Pathology International*.

[11] Franklyn J. A., Gammage M. D. (2007). Treatment of amiodarone-associated thyrotoxicosis. *Nature Clinical Practice Endocrinology and Metabolism*.

[12] Bartalena L., Brogioni S., Grasso L., Bogazzi F., Burelli A., Martino E. (1996). Treatment of amiodarone-induced thyrotoxicosis, a difficult challenge: results of a prospective study. *Journal of Clinical Endocrinology and Metabolism*.

[13] Houghton S. G., Farley D. R., Brennan M. D., Van Heerden J. A., Thompson G. B., Grant C. S. (2004). Surgical management of amiodarone-associated thyrotoxicosis:mayo clinic experience. *World Journal of Surgery*.

[14] Franzese C. B., Fan C. Y., Stack B. C. (2003). Surgical management of amiodarone-induced thyrotoxicosis. *Otolaryngology—Head and Neck Surgery*.

[15] Berti P., Materazzi G., Bogazzi F., Ambrosini C. E., Martino E., Miccoli P. (2007). Combination of minimally invasive thyroid surgery and local anesthesia associated to iopanoic acid for patients with amiodarone-induced thyrotoxicosis and severe cardiac disorders: a pilot study. *Langenbeck's Archives of Surgery*.

